# Assessment of the effect of *Schistosoma haematobium* co infection on malaria parasites and immune responses in rural populations in Gabon: study protocol

**DOI:** 10.1186/2193-1801-3-388

**Published:** 2014-07-29

**Authors:** Ulysse Ateba Ngoa, Jeannot Fréjus Zinsou, Roland Fabrice Kassa Kassa, Eliane Ngoune Feugap, Yabo Josiane Honkpehedji, Marguerite Massinga-Loembe, Hilaire Kenguele Moundounga, Anne Marie Nkoma Mouima, Lima Honorine Mbenkep, Linda Judith Wammes, Moustapha Mbow, Yvonne Kruize, Ghyslain Mombo-Ngoma, Aurore Larissa Bouyoukou Hounkpatin, Jean Claude Dejon Agobe, Issifou Saadou, Bertrand Lell, Hermelijn Smits, Peter Gottfried Kremsner, Maria Yazdanbakhsh, Ayola Akim Adegnika

**Affiliations:** Centre de Recherches Médicales de Lambaréné, BP: 118, Lambaréné, Gabon; Institut für Tropenmedizin, Universität Tübingen, Wilhelmstraβe 27, D-72074 Tübingen, Germany; Department of Parasitology, Leiden University Medical Center, Albinusdreef 2, 2333 ZA Leiden, The Netherlands; Faculté de Médecine, Université des Sciences de la Santé de Libreville, BP: 4009, Libreville, Gabon; Immunology Unit of the Laboratory of Bacteriology and Virology of Aristide Le Dantec Teaching Hospital, 30 Avenue Pasteur, BP 7325, Dakar, Senegal; Institute of Tropical Medicine of Antwerp, Nationalestraat 155, 2000 Antwerp, Belgium

**Keywords:** Malaria, Helminths, Co-infection, Cellular immune response, Study protocols, Epidemiology, Gabon, Africa

## Abstract

**Background:**

Malaria and helminth co infection are common in tropical and subtropical areas where they affect the life of millions of people. While both helminth and malaria parasites have immunomodulatory activities, little is known about the consequence of co-infections on malaria antigen specific immune responses.

**Method/Design:**

This study will be conducted in two rural areas of the Moyen Ogooué province in Gabon, endemic for both *Plasmodium falciparum* and *Schistosoma haematobium* infections. Participants, 5 to 50 years old, will be enrolled and grouped according to their infection status. *S. haematobium* and malaria parasites will be detected, demographic and clinical data will be recorded and blood will be collected for hematological as well as for immunological assays. The level of antibody specific to *Plasmodium falciparum* blood stage and gametocyte antigens will be measured using ELISA. PBMC will be isolated for phenotyping of different T cell subsets *ex vivo* by flow cytometry and for culture and cytokine response assessment.

**Discussion:**

We will provide a comprehensive picture of the interaction between schistosomes and malaria parasites which co-localize in peripheral blood. We will test the hypothesis that schistosome infection has an impact on specific humoral as well as on cellular immune responses to malaria antigens.

## Background

Malaria and helminth infections are two of the major causes of mortality and morbidity in developing countries (Brooker et al. 2007; Hotez and Kamath 2009; Mwangi et al. 2006). Both infections are highly endemic in tropical and sub tropical areas (Adegnika and Kremsner 2012; Akue et al. 2011; Brooker et al. 2007). In the tropics, Sub Saharan Africa (SSA) bears the heaviest burden of *Plasmodium spss.* infection occurring mainly in children under five years. Moreover it is reported that almost 90% of all schistosomiasis cases worldwide are confined into this part of the world (Hotez and Kamath 2009; Simoonga et al. 2009). In developing countries, infection with multiple species of parasites is often the norm (Griffiths et al. 2011; Raso et al. 2004).

Parasitic coinfection is a relatively new research area. Although some data have been generated, much is unknown and contradictions persist on the impact of helminth infections on malarial disease or parasitemia during co-infection (Adegnika and Kremsner 2012; Brooker et al. 2007; Hartgers and Yazdanbakhsh 2006; Nacher 2011). At the clinical level, interaction between plasmodium and helminth species has been discussed; while some studies have highlighted the protective effect of helminth infection on severe malaria and its association with a decreased incidence of malaria attacks or malaria parasite density (Boel et al. 2010; Lemaitre et al. 2014; Nacher et al. 2000), other studies have given a completely opposite picture (Le Hesran et al. 2004; Sangweme et al. 2010). It seems that the outcome of the interaction between helminth and malaria is helminth species specific with, for example, Ascaris infection more likely to be protective against severe forms of malaria and infection with hookworm associated with an increase of malaria incidence (Adegnika and Kremsner 2012; Nacher 2011). However despite these opinions more data are needed to get a clear picture of the situation.

Immunity and pathology to malaria is thought to be dependent on a balance between different arms of the immune system. Indeed, whereas at the early stages of infection, the presence of *Plasmodium spp.* in the blood stream is associated with the production of proinflammatory cytokines, activated cytotoxic T cells and γδ T cells, the effective clearance of the parasite is thought to be mediated by cytophilic antibodies of the IgG1 and IgG3 isotypes (Bouharoun-Tayoun and Druilhe 1992; Hartgers and Yazdanbakhsh 2006; Langhorne et al. 1998; Leoratti et al. 2008). However, the hallmark of immune responses during chronic helminth infections is the strong polarization toward Th2 and the downstream production of IgE and IgG4 antibodies. This Th2 skewed response is followed by the activation of an immunoregulatory network which can lead to cellular hyporesponsiveness with limited cells proliferation and cytokine production (Hartgers and Yazdanbakhsh 2006; Maizels and Yazdanbakhsh 2003; Nacher 2011). Down regulation of the immune response has been shown to be important for the survival of the parasite and for the restriction of deleterious immune response that lead to tissue pathology in the host (Belkaid 2007; Maizels and Smith 2011).

It is hypothesized that chronic helminth infections, with their marked immunomodulatory properties are able to modify immune responses to antigens derived from other pathogens (Hartgers and Yazdanbakhsh 2006; Maizels and Yazdanbakhsh 2003). This has been studied for helminth and malaria coinfection but again with conflicting results. For example, studies reported that schistosome infections decrease (Courtin et al. 2011) or favor (Remoue et al. 2003) the production of cytophilic antibodies protective against *Plasmodium falciparum* malaria, while another study in Zimbabwe, reported no association between *Schistosoma* infection and humoral response to malaria parasites (Sangweme et al. 2010). Inconclusive results were also reported when assessing cytokine productions in malaria co-infected subjects (Sangweme et al. 2010). In two different studies undertaken in Ghana and Mali, IL-10 responses to malaria antigen were found to be higher in helminth and malaria co-infected subjects (Hartgers et al. 2009; Lyke et al. 2012) whereas in a study from Senegal the level of INFγ was higher in co-infected subjects and the increase of IL-10 was only observed in adults but not in children when considering schistosoma and malaria co-infection (Diallo et al. 2004). Helminth infections have also been thought to increase malaria transmission intensity (MTI), as demonstrated in two studies showing an increase of gametocyte carriage in helminth infected subjects in comparison to non-infected ones (Nacher et al. 2001; Sangweme et al. 2010). Interestingly, using a murine model of malaria and helminth coinfection, Noland *et al.* observed that anopheles mosquitoes exposed to co-infected mice had a higher rate of infectivity than those exposed to malaria only infected mice (Noland et al. 2007). The mechanism behind the possible impact of helminths on malaria transmission is still unclear.

Taking into account the reports in literature, we set out to conduct a study in an area where *S. haematobium* and *P. falciparum* infections are highly endemic (Adegnika et al. 2010; Wildling et al. 1995). The study will test the hypothesis that active helminth infections alter the humoral and cellular immune response to *Plasmodium falciparum* antigens. A global picture of both the antibody signature and the cytokine profiles will be obtained as well as the activation status of B Cells, T cells, monocytes and dendritic cells. Furthermore the role of regulatory T cells will be assessed by functional analysis, using Treg depletion strategies and comparison of immune responses to depleted and Treg containing cell fractions.

## Methods/design

### Study site

**Figure 1:**
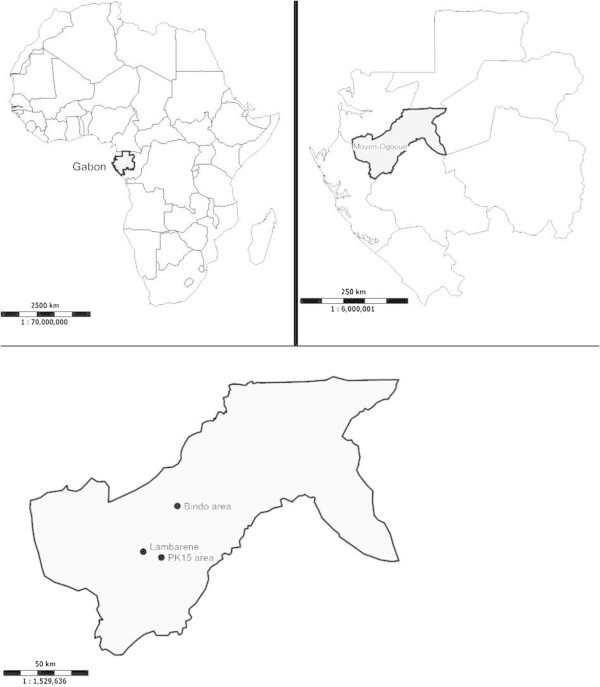
**Map representing the localization of the study area (lower panel).** The upper panel shows the emplacement of Gabon in Africa (in the left) and the localization of the Moyen Ogooué province in Gabon (in the right).

The study has taken place in two distinct areas, the Bindo village and the Pk15 area, located in the Moyen-Ogooué province of Gabon, central Africa. Several lakes are present in this district and the temperature has an average of 27°C. The capital of the Moyen-Ogooué province is Lambaréné, a semi urban town of about 35.000 inhabitants surrounded by villages. Bindo and the Pk15 are respectively 60 and 15 kilometers from Lambaréné (Figure 1) and they similarly present all the characteristics of a rural area.

Bindo village is a relatively remote place with around 1000 inhabitants. It is located in the center of a palm tree plantation. The income is generated mainly from work at the palm plantation owned by a private company. Few inhabitants get their income from fishing activities and almost all of the population grows their own food. The water is supplied directly from the Ogooué River to two public taps in the village through a pipeline. The village has one nursery, one primary school and one small shop which sell manufactured products.

In contrast to Bindo, the Pk15 area is closer to Lambaréné; it is stretched along a national single lane highway over a 30 km distance. The population is very mobile and due to the proximity to Lambaréné the source of the income is diverse although most of the inhabitants work in an industrial palm oil plantation and engage in agriculture and fishing activities for their subsistence. Four primary schools and two nurseries are present in the Pk15 area. The area has neither electricity nor tap water and drinkable water is taken from streams neighboring the houses.

The streams both in the Pk15 area and Bindo represent the main source of drinking water. They are the sites for laundry and bathing but also represent important points for contact with *S. haematobium* which has been observed to be highly prevalent in these areas. Furthermore, numerous studies have shown that malaria, intestinal helminth and filarial infections are prevalent in these areas (Adegnika et al. 2010; Wildling et al. 1995).

### Study design, study population and ethical issues

The study design is cross sectional, aiming to assess interaction between helminth and malaria infections. Ethical clearance was obtained from the regional ethic committee of Lambaréné (CERIL). Prior to enrolment, the protocol was explained to each participant or to their legal parent or guardian if they were under 18 years of age and a written informed consent was obtained. The study was preceded by a pilot phase so as to set up the study procedures and better characterize the study area and the study population. To be eligible for the study, subjects had to be between 5 and 50 years of age and living in the study area for at least one year. We did not include subjects with a hemoglobin level below 8 g/dl or with known HIV infection. In addition, intake of praziquantel less than one year prior to the study was also a non-inclusion criterion.

### Recruitment of participants, data and samples collection

Local authorities and villagers were informed about the study through meetings and home visits. A parasitological laboratory was set up in the Bindo area by the study team in order to process the samples on time during the study. The relative proximity of the Pk15 area to Lambaréné meant that samples for parasitological diagnosis could be processed in the main laboratory in Lambaréné.

After informing the communities, all the houses in both villages were identified and geographical coordinates were taken to allow mapping of the study area by global positioning system (GPS). Villagers were then visited at home by field workers. During these visits the study was explained in detail to the occupants, questions regarding the study were answered and people willing to participate and who were eligible were enrolled.

Following the informed consent process a questionnaire was administered to each participant or their parents in case they were unable to answer, to collect demographic and health history data. For all eligible participants a clinical examination was performed to determine the presence of hepatomegaly and/or splenomegaly as well as other relevant health conditions, urine samples were obtained for detection of *S. haematobium* infection and blood was drawn for parasitological and immunological analysis as described in sections below. All the data collected during the study was on paper forms which were then entered into OpenClinical, clinical trial software for electronic data capture.

### Parasitological examination

#### Detection of Schistosoma haematobium eggs

Presence of *S. haematobium* eggs was detected microscopically in 10 ml of fresh urine passed through a filter of 12-mm pore-size (Millipore).

A subject was classified as free of infection if no *S. haematobium* egg was detected in three samples of urine collected in three consecutive days. Any subject with at least one *S. haematobium* egg found in the urine was classified as infected.

#### Detection of Plasmodium spp. blood stage parasite

*Plasmodium spp.* infection was assessed by microscopic examination. Presence of asexual form of the parasite will be determined using the Lambaréné method as described elsewhere (Kremsner et al. 1988; Planche et al. 2001). On the other hand presence of sexual form of the parasite will be established by the WHO method after a minimum count of 1000 white blood cells.

Real time-PCR will be performed on DNA extracted from EDTA blood pellet kept frozen at minus 80°C. This will be done in addition to microscopy examination to increase the sensitivity of parasites detection. A detailed description of the procedure has been published elsewhere (Adegnika et al. 2006).

#### Detection of microfilaria in blood

Microfilaria species in blood will be detected by leucoconcentration and microscopy using a modified Knott’s technique (Goldsmid 1970). One milliliter of blood will be collected in an EDTA tube and dispensed in an equal volume of 2% saponin lysing solution. After centrifugation the sediment will be transferred to a slide. The entire slide will be examined using a microscope and the number of microfilaria will be counted. Differentiation between *Loa loa* and *Mansonela spp*. will be based on the identification of the sheath of *Loa loa* after the addition of one drop of methylene blue to the slide.

### Immunological analysis

For immunological assays blood will be drawn in the field from the participants and will be brought to the laboratory facilities within 6 hours. In order to address the research questions immunological assays including antibody measurements by ELISA, whole blood culture assays, human peripheral mononuclear cells (PBMC) isolation and stimulations, cytokine production analysis by flow cytometry (through intracellular staining) and multiplex bead analysis (released cytokines in supernatants measured by luminex), and gene expression by multiplex analysis will be performed as described below.

#### Humoral immunological assays

We will measure total IgG specific to asexual and sexual forms of *P. falciparum* by ELISA as previously described (Ouédraogo et al. 2011). Antibody response to the apical membrane antigen 1 (AMA-1), merozoite surface protein 1_19_ (MSP-1_19_) and glutamate rich protein (GLURP) antigens will be used as a markers of cumulative exposure to *P. falciparum*. On the other hand total IgG to Pfs48/45 and Pfs230 will be measured to determine *P. falciparum* gametocyte carriage and antibody response over time as described earlier (Bousema et al. 2006).

#### Cellular immunological assays

##### Media preparation

For the immunological essays media will be prepared as described in below.

RPMI-S1 medium: RPMI-1640 (Invitrogen, Breda, The Netherlands) supplemented with 100 U/ml penicillin (Astellas Pharma B.V.), 100 μg/ml streptomycin (Sigma-Aldrich, Zwijndrecht, The Netherlands), 1 mM pyruvate (Sigma-Aldrich, Zwijndrecht, The Netherlands) and 2 mM L-glutamine (Sigma-Aldrich, Zwijndrecht, The Netherlands).

RPMI-S2 medium: RPMI-1640 (Invitrogen, Breda, The Netherlands) supplemented with 20% Fetal Calf Serum (FCS, Greiner Bio-One, Alphen a/d Rijn, The Netherlands), 100 U/ml penicillin and 100 μg/ml streptomycin.

RPMI-S3 medium: RPMI-1640 supplemented with 10% FCS, 100 U/ml penicillin and 100 μg/ml streptomycin.

IMDM-S1 medium: IMDM (Invitrogen, Breda, The Netherlands) supplemented with 20% FCS, 100 U/ml penicillin, 100 μg/ml streptomycin, 1 mM pyruvate and 2 mM L-glutamine).

IMDM-S2 medium: IMDM supplemented with 10% FCS,100 U/ml penicillin, 100 μg/ml streptomycin, 1 mM pyruvate and 2 mM L-glutamine.

Freezing medium: RPMI-1640 + 20%FCS supplemented with 20% Dimethyl sulfoxide (DMSO, Merck KGaA, Darmstadt, Germany).

FACS buffer: 500 ml PBS (Invitrogen, Breda, The Netherlands), 0.5% BSA (Roche Diagnostics GmbH, Mannheim, Germany), 2 mM EDTA (Sigma-Aldrich, Zwijndrecht, The Netherlands) and 2 ml of 0.5 M stock).

FACS staining solution: FACS buffer with 1% human FcgR-binding inhibitor (eBioscience, San Diego, CA, USA).

Permeabilization buffer: FACS buffer with 0.5% saponin (Sigma-Aldrich, Zwijndrecht, The Netherlands).

##### Whole blood culture

Heparinized blood will be diluted in equal volume of RPMI-S1 medium and cultured in 96 wells round bottom plates. Hundred micro liters of diluted blood will be distributed in each well and cultured with 100 μl of RPMI-S1 medium and with one of the following stimuli: CPG (5 μg/ml, Cayla-Invivogene Europe, Toulouse, France), LPS (100 ngml, Cayla-Invivogene Europe, Toulouse, France), LPS + CPG, schistosoma eggs antigens (SEA, 10 μg/ml, prepared by the Leiden University Medical center, The Netherlands (LUMC)), adult worm antigen (AWA, 10 μg/ml, prepared by the Leiden University Medical center, The Netherlands (LUMC)), PAM3 (100 ng/ml, EMC Microcollection GmbH, Tübingen, Germany), FSL1 (100 ng/ml, Cayla-Invivogene Europe, Toulouse, France), CLO97 (1 μg/ml, Cayla-Invivogene Europe, Toulouse, France). After 24 hours incubation at 37°C, supernatants will be collected, split into 2 tubes and kept at -20°C until further analysis.

##### PBMC isolation

For this assay, peripheral blood will be collected in sodium heparinized tubes (BD, Franklin Lakes, NJ, USA). PBMC will be isolated from blood by density gradient centrifugation on Ficoll (Apotheek AZL, Leiden, The Netherlands) as described earlier (Yazdanbakhsh et al. 1993). We expect an average of 25 × 10^6^ PBMCs per donor, which will be used for the different assays planned, as follows in sections below.

##### Cryopreservation of PBMC

PBMC will be resuspended at the concentration of 10 × 10^6^ in a solution of RPMI-S2 without glutamax. An equal volume of a freezing medium will then be added to the cell suspension. Finally the total amount of cells will be split into cryovials. One milliliter of the cells suspension containing 5 × 10^6^ PBMC will be transferred in each cryovial. All the cryovials will be put in a Mr Frosty and will be kept overnight in a -80° freezer and transferred into a liquid nitrogen containing tank the next day for long term storage.

##### Thawing and resting of cryopreserved cells

Cryovials will be collected from the Liquid nitrogen tank and defrosted in a 37°C water bath. For each cryovial the cell suspension is transferred into a corresponding 50 ml conical tube. PBMC will then be washed two times with RPMI-S3 without glutamax and resuspended at the concentration of 0.5 to 2 × 10^6^ cells/ml in RPMI-S3 medium. PBMC will finally be allowed to rest for 4–6 hour in a 5% CO_2_ incubator at 37°C.

##### PMA/Ionomycin stimulation of PBMC for intracellular cytokine measurement

For this assay 3.5 × 10^6^ PBMC will be transferred to a 5 ml tube and washed with 2 ml of IMDM-S2 medium. The suspension will be spun for 5 minutes at 20°C, 1500 rpm. Supernatant will then be discarded and the cells will be resuspended in 300 μl of IMDM-S1. Hundred micro liter of the cells suspension (1 × 10^6^) will be cultured in 96 wells round bottom plate with PMA/Ionomycin (100 ng/ml and 1 μg/ml respectively, Sigma Aldrich), SEB (10 μg/ml, Sigma-Aldrich, Zwijndrecht, The Netherlands) or medium as negative control. After 2 hours incubations at 37°C, 4 μg/ml of Brefeldin A (Sigma-Aldrich, Zwijndrecht, The Netherlands) will be added to each well and cells will be incubated for 4 additional hours at 37°C before fixation by 1.9% paraformaldehyde (PFA) fixative (Sigma-Aldrich, Zwijndrecht, The Netherlands).

##### **PBMC depletion of CD4**^**+**^**CD25**^**high**^**regulatory T cells**

Depletion of PBMC from CD4^+^CD25^high^ regulatory T cells (Treg) will aim to assess how Treg cell function is affected during helminth and malaria coinfection. Total and Treg cell-depleted PBMC will be stimulated by different antigens or mitogens and their response will be compared. A total of 12 × 10^6^ PBMC will be needed for this experiment. Treg cells will be isolated by MACS using the CD4^+^CD25^high^ regulatory T cells kit (Miltenyi Biotec GmBH, Bergisch Gladbach, Germany). As already described cells isolation will be done in a two-step procedure (Wammes et al. 2012). Briefly, CD4^+^ cells will be enriched by negative selection and then labeled with CD25^+^ microbeads for a subsequent selection of CD4^+^CD25^+^ regulatory T cells. It should be noted that both depleted and whole PBMC cell populations will undergo identical procedure involving the MACS columns but in the latter population, CD4^+^CD25^+^ regulatory T cells will be added back (mock depletion).

##### **Proliferation assay of total and CD4**^**+**^**CD25**^**high**^**regulatory T cells depleted PBMC**

This assay will be performed using the green-fluorescent dye carboxyfluorescein succinimidyl ester (CFSE, Sigma-Aldrich, Zwijndrecht, The Netherlands) so as to follow cells proliferation (Quah et al. 2007). To load the cells with CFSE, total and depleted PBMC will be resuspended in PBS (Invitrogen, Breda, The Netherlands) at a concentration of 2 × 10^7^ cells/ml. CFSE will then be added at the concentration of 2 μM and cells will be incubated for 15 minutes at room temperature in the dark. After the incubation time, CFSE staining will be stopped by adding 4 ml of IMDM-S2 medium for 1 minute. Finally, cells will be spun down for 5 min 1800 rpm 20°C and the supernatant will be decanted. The loaded cells will then be ready for culture.

##### **Antigen stimulation of total and CD4**^**+**^**CD25**^**high**^**regulatory T cells depleted PBMC**

CFSE-labeled Treg cell-depleted and undepleted PBMC, will be cultured in a 96 wells round bottom plates. Cells will be seeded at the concentration of 4 × 10^5^ cells and cultured for 3 days with 100 μl of malaria infected red blood cells (iRBC; 1 × 10^6^ cells per well prepared by the Leiden University Medical center, The Netherlands (LUMC)), malaria uninfected red blood cells (uRBC; 1 × 10^6^ cells per well prepared by the Leiden University Medical center, The Netherlands), SEB (10 μg/ml), PPD (10 μg/ml, Statens Serum Institute, Denmark, Copenhagen), SEA (20 μg/ml), AWA (10 μg/ml). At day 3 of culture, supernatants will be collected and keep at -20°C for cytokine measurements by luminex or ELISA. In addition, cells will be harvested, fixed with 1,9% PFA for 15 min and stored at -80°C until further analysis for cell division detected by CSFE labeling using flow cytometry.

#### Cytokine production analysis

Cytokine production will be measured in the supernatants obtained, after three days stimulation of total and CD4^+^CD25^high^ regulatory T cells depleted PBMC and 24 hours of whole blood culture. Cytokine levels will be measured using the multiplex beads array immunoassay or ELISA according to standard procedure. A quantification of different cytokines will be done in two different panels to characterize the cytokine profile involved in innate immunity (IFNa2, IL1b, IL6, IL10, IL12p70, IL-13, IL-23, IFNg, MCP1, MIP1a, MIP1b, TNFa and IP-10) and adaptive immunity (TNF-a, IFN-g, IL-2, IL-4, IL-5, IL-13, IL-17A, IL-17 F, IL-22, IL-10, and IL-21) respectively. The multiplex beads kit will be obtained from Bio-Rad Laboratories and samples will be acquired on the Bio-Plex 200 system (Bio-Rad Laboratories) following the manufacturer recommendations. The most informative cytokines will be used for measurement of further samples.

#### FACS analysis

##### Cell surface marker staining

Cells immunophenotyping will be performed by flow cytometry. Cells will be seeded at the concentration of 2 × 10^5^ to 4 × 10^5^ cells per well in a 96 well FACS V-bottom plates and resuspended in FACS buffer. Extracellular staining of the cells will be done by a mixture of all the fluorescent labeled antibodies of interest prepared in a FACS staining solution at optimal working concentration. After centrifugation and discarding of FACS buffer, cells will be resuspended in 30 μl of the antibody solution and incubated for 30 minutes in the dark at 4°C. Following this step, 100 μl of FACS buffer will be added to each well, cells will be spun again and supernatant discarded. Finally the labeled cells will be resuspended in 50 μl of FACS buffer for acquisition. Acquisition will be performed on a FACS calibur flow cytometer (Becton Dickinson Biosciences BD) and data will be analysed by Flowjo software (Treestar Inc., Ashland, OR, USA). We will use different panels of fluorescently labeled antibodies and cluster various differentiation markers to be able to identify B cells, DCs, monocytes and different T cells subsets and other interesting cells of the immune system such as natural killer (NK) cells or γδ T cells that have recently been shown to be associated with immune response against malaria.

##### Intracellular staining

To allow the assessment of cell-specific cytokine production, intracellular cytokines in combination with various subset markers for dendritic cells (DCs), B cells, monocytes and/or (γδ) T cells will be labeled by fluorescent antibodies. Staining of intracellular cytokines will be performed by a two step procedure consisting of permeabilization and staining of the cells. For PFA fixed cells permeabilization will be performed using a permeabilization buffer. For intracellular staining of FoxP3, PBMC will be fixed and permeabilized by a FoxP3 fixation and permeabilization kit (eBioscience, San Diego, CA, USA). FACS staining will be done as described in the previous paragraph with one additional washing step with permeabilization buffer before adding the antibody mix.

#### Innate gene profiling

Gene profiling will be performed by Reverse Transcription Multiplex Ligation-dependent Probe Amplification (RT-MLPA) in a subset of the study participants. A volume of 2.5 ml of blood will be collected in a PAXgene Blood RNA tube (PreAnalytiX, Quiagen, Germany) per subject. Gene expression profiling of various pattern recognition receptors (PRRs) as well as several key cytokines and chemokines (CCL2; 5 and 22, CXCL13, IL-10, IL-12p40/35, IL-23p19) will be done as detailed previously (Joosten et al. 2012) to assess whether cells signaling and trafficking is differentially affected in single infection versus coinfection.

### Analysis plan

The aim of this project is a) to determine how concurrent schistosome infections can affect malaria infections and malaria transmission intensity. Specifically we will assess how levels antibodies to asexual and gametocyte antigens, marker of gametocytogenesis are influenced by concurrent schistosome infection and b) to assess how general and malaria-specific immune responses are modified by concurrent schistosome infection. By general we mean responses to mitogens and activation status of immune cells. On the other hand by malaria specific-immune response we refer to cytokine production and activation in response to infected red blood cells. This study will address the following research questions:

- What is the impact of *S. haematobium* infection on malaria transmission: how does it impact gametocyte carriage and humoral response to gametocytes?
- What is the level of pro-inflammatory and anti-inflammatory cytokines during malaria and/or schistosome infection in response to stimuli that activate the innate and adaptive immune system?
- Are the phenotype of T cells, B cells, DCs and monocytes different during malaria and/or schistosome infection in general, and in an antigen specific manner, in particular?
- What is the functional capacity of CD4^+^CD25^high^regulatory T cells, as assessed by depletion on the immune response in case of malaria and/or schistosome infection?
- Is the gene expression signature of the immune system altered during schistosoma and malaria coinfection?

These questions will be answered by considering the two study outcomes represented first by the infectious status of participants and secondly by the immune response. For this cross sectional study, four groups will be compared; Plasmodium spp. and S. haematobium uninfected, *Plasmodium spp.* and *S. haematobium* coinfected, *Plasmodium spp.* only infected as well as *S. haematobium* only infected group. Baseline characteristics will be determined and differences of cellular immune response between groups will be assessed.

### Sample size calculation

For detailed immunological studies the sample size calculation was based on the proportion of responders between infected versus uninfected participants (either malaria or schistosomiasis infection). Considering previous unpublished data we hypothesized that the proportion of responders with regard to the levels of CD4^+^CD25^high^FoxP3^+^ T cells will be 15% in the uninfected group and 50% in the infected group necessitating a sample size of 28 subjects per group (power = 0.80 and p = 0.05). To correct for an estimated 10% failure rate in sample processing we included 31 subjects per group. This sample size will permit to measure other outcomes like difference in cytokine production with adequate power. For example the study will have a power of 0.7 to see an increase in IL10 responders from 30 to 60%, which we expect from schistosomiasis carriers (Hartgers et al. 2009; Meurs et al. 2011; van der Kleij et al. 2004).

On the other hand the above calculated sample size only gives a power of 0.32 to detect an effect of helminth infection on antibody response to sexual or asexual stage antigens. Therefore we run an additional sample size calculation that was based on data gathered from an immuno epidemiological study conducted in a setting similar to our study site. From this study it appears that gametocyte antigens usually elicit a weaker antibody response than asexual stage antigens and that the percentage of IgG responders to Pfs48/45 and Pfs230 was around 22 to 28% (Ouédraogo et al. 2011). Given the fact that we did not have data comparing antibody response specific to Pfs48/45 and Pfs230 between schistosoma infected and uninfected subjects, we hypothesize that helminth infection will lead to a maximum twofold change in antibody response to Pfs48/45 and Pfs230. Considering this we calculated that a sample size of 63 subjects per group (power = 0.90 and p = 0.05) would be adequate to answer our research question. Based on the literature it antibody response to sexual or asexual stage antigens may vary according to the age of the subjects. Hence as our study will include both children and adult our final sample size will be of 126 subjects per group.

## Results from pilot study

**Figure 2:**
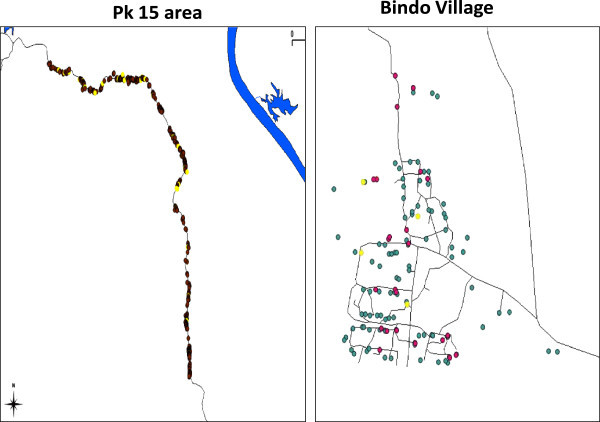
**Mapping of the PK15 area and the Bindo village.** Each dot represents a single house. In the left panel the yellow dots indicate houses where *Schistosoma haematobium* infected subjects have been found during the screening phase. In the second panel the green dots represent houses not selected for the screening phase whereas the yellow and the red dots respectively represent houses where Schistosoma infected and uninfected subjects were living.

**Figure 3:**
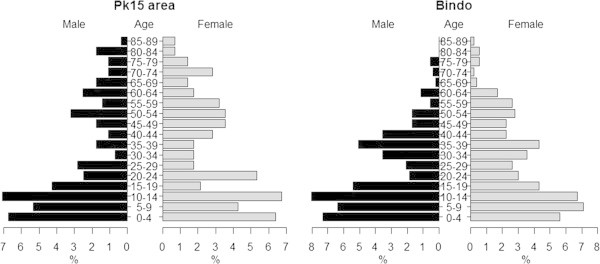
**Age pyramids of the population of the Pk15 area and the Bindo village.**

**Figure 4:**
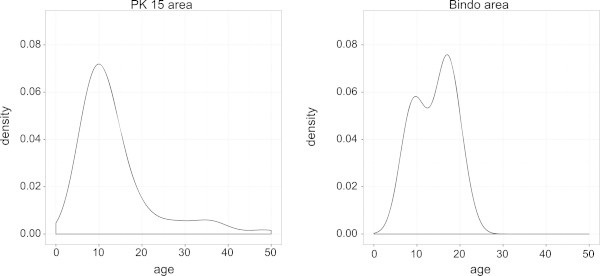
**Kernel density estimation of** ***Schistosoma haemtobium*** **infection per age in the the Pk15 area and the Bindo village.** This estimation is based on results of a random sample of around 10% of the population of both area.

**Figure 5:**
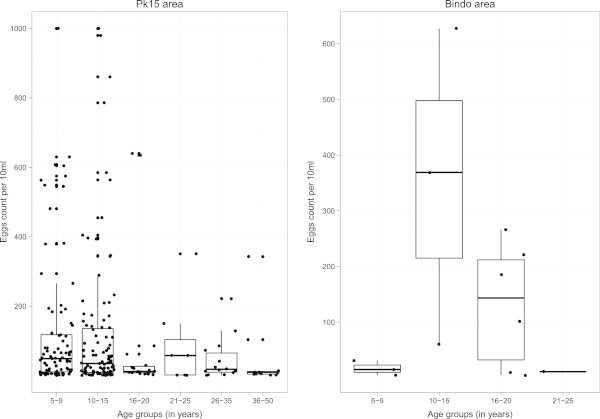
**Intensity of** ***Schistosoma haematobium*** **infection per age group in the different study area.** Boxplot represent the median eggs count (median horizontal line) as well as the first (lower horizontal line) and the third (upper horizontal line) interquartile. Each dot is representative of the eggs count of a single subject.

Pilot studies have been conducted in the PK15 area and the Bindo village to characterize the study population and to set up the study procedure. All houses in both areas have been identified and their GPS coordinates recorded (Figure 2). To obtain demographic data and to establish the epidemiological feature of *S. haematobium* infection in the study area a random selection of houses has been screened to represent around 10% of the population. Typical to low income countries the age pyramid curve presented a broad base as shown in Figure 3. In both areas the age pyramids show that only few adults from 20 to 49 are living in the village. This could be due to the migration of young adults to big cities for work or for study purposes. In Bindo this is less apparent as inhabitants are employed by the palm oil company. Regarding the *S. haematobium* infection we found an overall prevalence of 43% in the PK15 area and 15% in Bindo village. This difference between the two areas could be explained by the fact that in the Pk15 area streams represent the first source of water compared to the Bindo village where public water pumps are available. As represented in the age prevalence curve, children were the most infected by *S. haematobium* and showed the highest infection intensity (Figures 4 and 5).

## Conclusion

Polyparasitism is usually described as common in tropical and sub tropical areas where the majority of the population is simultaneously exposed to various pathogens. Malaria and helminth coinfection is very common and it is important to understand their interaction. So far only few studies have been conducted on the interaction between these two pathogens and their effect on the human host in much detail. Moreover, the data generated so far provide contradictory results. At the immunological level, the data available has shown that chronic helminthiasis can modulate and impair immune responses specific to malaria antigens*.* It has been proposed that modulation may rely on two mechanisms. Firstly, through the skewing of cytokine production toward a Th2, which could alter the antibody isotypes and cellular responses generated against malaria parasites. Secondly, by the induction of regulatory cells which indue a hypo responsiveness milieu impairing the cellular response against *Plasmodium*. Despite this general assumption that helminth infections modulate malaria immunity, many questions need to be answered in great detail to understand and unequivocally establish whether there is an interaction between these two parasites. One of them concerns the effect of chronic helminth infection on cell specific immune responses in terms of CD4^+^CD25^high^ Treg cells and the control of other cytokine producing effector (T) cells. What is new in this study is the aim to unravel general patterns in innate and adaptive immune responses both at the cellular and molecular level, providing insight in innate immunity shifts that precede and dictate adaptive immune responses in single or co-infected individuals. The identification of malaria responsive cell subsets is needed; the contribution of DCs, monocytes, CD4 or CD8 T cells, γδ T cells or NK cells to malaria antigen-specific responses and the influence of helminth infections on these subsets will be studied.

These studies will shed light on the possible interaction between schistosome and malaria parasites and pave the way for future interventional studies.
